# Convenient methodology for extraction and subsequent selective propagation of mouse melanocytes in culture from adult mouse skin tissue

**DOI:** 10.1016/j.bbrep.2019.100619

**Published:** 2019-02-22

**Authors:** Nahoko Tomonobu, Rie Kinoshita, I. Wayan Sumardika, Youyi Chen, Yusuke Inoue, Akira Yamauchi, Ken-ichi Yamamoto, Hitoshi Murata, Masakiyo Sakaguchi

**Affiliations:** a Department of Cell Biology, Okayama University Graduate School of Medicine, Dentistry and Pharmaceutical Sciences, Okayama, Japan; b Faculty of Science and Technology, Division of Molecular Science, Gunma University, Kiryu, Gunma, Japan; c Department of Biochemistry, Kawasaki Medical School, Kurashiki, Okayama, Japan; d Faculty of Medicine, Udayana University, Denpasar, Bali, Indonesia

**Keywords:** Melanocytes, Melanoma, Metastasis, Primary culture, MCAM, melanoma cell adhesion molecule, TPA, 12-O-Tetradecanoylphorbol 13-acetate, TRP-1, tyrosinase-related protein-1, αSMA, alpha smooth muscle actin

## Abstract

Mouse melanoma B16-BL6 cells are useful cells for cancer metastatic studies. To understand the metastatic principle at molecular levels, it is necessary to carry out experiments in which cancer cells and their normal counterparts are compared. However, unlike normal human melanocytes, preparation of normal mouse melanocytes is quite difficult due to the lack of marketing and insufficient information on an established protocol for primary culture of mouse melanocytes. In this study, we aimed to establish a convenient method for primary culture of mouse melanocytes on the basis of the protocol for human melanocytes. The main obstacles to preparing pure mouse melanocytes are how to digest mouse skin tissue and how to reduce the contamination of keratinocytes and fibroblasts. The obstacles were overcome by collagenase digestion for skin specimens, short time trypsinization for separating melanocytes and keratinocytes, and use of 12-O-Tetradecanoylphorbol 13-acetate (TPA) and cholera toxin in the culture medium. These supplements act to prevent the proliferation of keratinocytes and fibroblasts, respectively. The convenient procedure enabled us to prepare a pure culture of normal mouse melanocytes. Using enriched normal mouse melanocytes and cancerous B16-BL6 cells, we compared the expression levels of melanoma cell adhesion molecule (MCAM), an important membrane protein for melanoma metastasis, in the cells. The results showed markedly higher expression of MCAM in B16-BL6 cells than in normal mouse melanocytes.

## Highlights

• Collagenase efficiently extracts skin cells.
• Short trypsinization greatly helps an enrichment of mouse melanocytes in culture.

## 1 Introduction

Normal cells in cultivation are a crucial material in experimental studies in the field of life science and its relevant fields, especially in comparison with their abnormal counterparts such as cancer cells, by which the causes of the alteration or changing events can be determined at both cellular and molecular levels. We have been conducting mechanistic studies on lung tropic melanoma metastasis [1,2], and we have found that S100A8/A9, a heterodimer complex of S100A8 and S100A9 proteins [[3], [4], [5]], which are Ca2+ binding small proteins of about 10 KDa in molecular mass belonging to the S100 family, and its novel receptor, melanoma cell adhesion molecule (MCAM) play important roles in the metastasis [6,7]. Owing to the intrinsically different characters of cancer cells from the normal counterparts in our living body, the lung, one of the very sensitive tissues to cancer cells as a foreign substance, falls into a state of cancer-derived inflammation, resulting in the production and secretion of S100A8/A9 there at a significant level [8,9]. On the other hand, distant melanoma cells catch the S100A8/A9 signal from the inflammatory lung through the MCAM sensor that exists on the melanoma cell surface, resulting in acceleration of lung-oriented metastasis of melanoma cells. This metastatic event *in vivo* was observed in a well-established syngenic model using mouse B16-BL6 melanoma cells and immunocompetent C57BL/6J mice [6]. In this system, human melanoma cells are not adapted because of immune exclusion of human cells. To understand the metastatic role of MCAM in mouse B16-BL6 melanoma cells, it is inevitably required to learn expression level of MCAM in mouse B16-BL6 melanoma cells in comparison to that in its normal counterparts. However, we faced a difficult problem in the preparation of normal mouse melanocytes at that time. Surprisingly, unlike normal human melanocytes, normal mouse melanocytes were not marketed widely as a commercial product, and little is known about the methods for isolation and cultivation of normal mouse melanocytes. This is probably due to technically difficult problems for effective isolation of cells with maintenance in a living condition and subsequent selective propagation of a melanocyte population from the adult mouse skin tissue since distributions of melanocytes in the skin of mice and humans are different.

We confirmed that the expression level of MCAM was highly elevated in various human melanoma cell lines in a consistent manner when compared to that of normal human melanocytes from a commercial source (our unpublished data). However, at that time, we could not define the expression level of MCAM protein in mouse melanoma cell lines in comparison to their normal counterparts. We therefore tried to establish a convenient method to readily extract and selectively propagate a normal mouse melanocyte population from adult mouse skin tissue. When the isolated melanocytes were eventually compared with B16-BL6 melanoma cells for their intrinsic MCAM expression, we confirmed that MCAM shows markedly higher expression at the protein level in B16-BL6 melanoma cells than in normal mouse melanocytes.

## 2 Materials and methods

### 2.1 Cell lines

B16-BL6 cells (a highly invasive variant of the mouse malignant melanoma B16 cell line; kind gift from Dr. Isaiah J. Fidler, M. D. Anderson Cancer Center, Houston, TX) were cultivated in D/F medium (Thermo Fisher Scientific, Waltham, MA) supplemented with 10% FBS in a humidified incubator. B16-BL6 cell culture was checked for mycoplasma by using a mycoplasma detection kit (Thermo Fisher Scientific) and Hoechst 33342 staining at regular intervals of time.

### 2.2 Normal mouse melanocytes

**Fig. 1:**
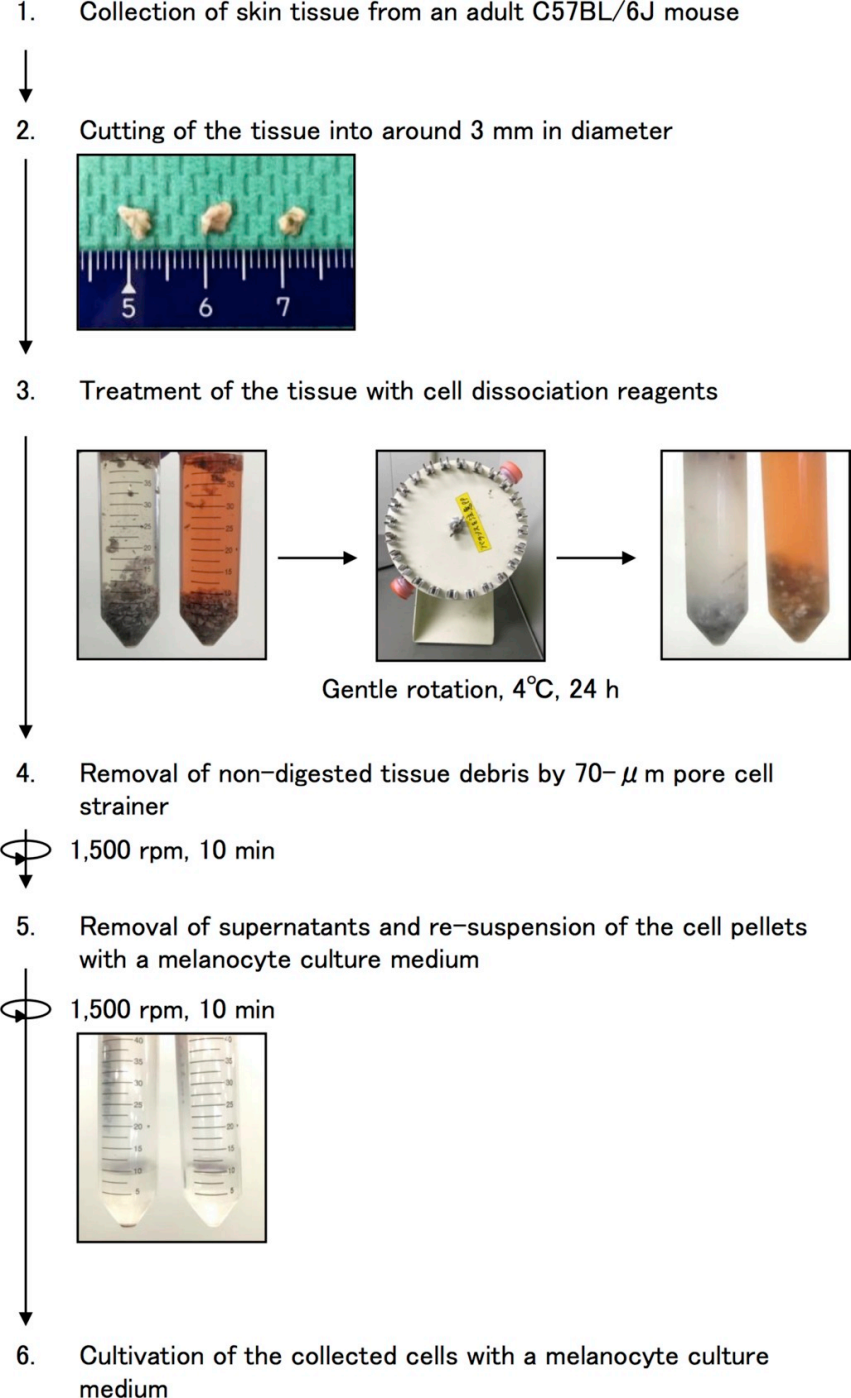
Procedure for extraction of melanocytes from mouse skin tissue and subsequent selective propagation in culture. The details are shown in materials and methods.

Skin tissue was collected from an 8-week-old C57BL/6J mouse after epilation and chopped into pieces of about 3 mm in diameter (see Fig. 1). The collected tissues were then treated with either a serum-free D/F medium (Thermo Fisher Scientific) containing collagenase (WAKO, Hiroshima, Osaka, Japan) at a final concentration of 1 mg/ml or a serum-free trypsin medium (TrypLE™ Express, Thermo Fisher Scientific), both media supplemented with kanamycin (50 μg/ml) and amphotericin B (100 μg/ml), for 24 h at 4 °C under gentle rotation. After incubation of the specimens, tissue debris was removed by passing the mixture through a 70-μm pore sized cell strainer (Corning, Corning, NY). The collected cell suspensions were centrifuged at 1500 rpm for 10 min, and the clear supernatants were removed. Then a melanocyte culture medium (a modified medium on the basis of the DermaLife Ma Melanocyte Medium Complete Kit; Lifeline Cell Technology, Frederick, MD) supplemented with 12-O-Tetradecanoylphorbol 13-acetate (TPA, 10 ng/ml, WAKO) and cholera toxin (10 nM, Sigma-Ardrich, St. Louis, MO) was added. At this time, the epidermal cell mixtures in pellets were disaggregated mechanically by repeated pipetting up and down and were seeded on a culture dish (35 mm in diameter). The culture medium was changed after 48 h and kept for another 3 days. When the cell density had reached about 70% confluency, the cells were subcultured by trypsinization with 0.05% trypsin/0.02% EDTA solution at room temperature. To collect as many melanocytes as possible, trypsinization was done shortly under microscopically checking the state of melanocyte detachment that sets apart from that of keratinocyte detachment. The cells were then continuously cultivated.

### 2.3 Western blot analysis

Western blot analysis was performed under conventional conditions. The antibodies used were as follows: rabbit anti-MCAM antibody (Sigma-Aldrich, St Louis, MO), mouse anti-TRP1 antibody (Santa Cruz Biotechnology, Santa Cruz, CA), rabbit anti-cyclin D1 antibody (Cell Signaling Technology, Beverly, MA), mouse anti-cyclin D3 antibody (Cell Signaling Technology), rabbit anti-cyclin E1 antibody (Cell Signaling Technology), mouse anti-p21/WAF1 antibody (Merck KGaA, Darmstadt, Germany) and mouse anti-tubulin antibody (Sigma-Aldrich). The second antibody was horseradish peroxidase-conjugated anti-mouse or anti-rabbit IgG antibody (Cell Signaling Technology). All primary antibodies used show cross-reactivity to their targeted proteins from not only human but also mouse source.

## 3 Results and discussion

### 3.1 Extraction of skin cells from adult mouse skin tissue

In human skin, simple enzymatic digestion using trypsin is sufficient to dissociate melanocytes from a skin specimen since human dermal melanocytes are mainly located in the basal layer of the skin epidermis [10,11]. However, a trypsin method similar to that used for human melanocytes may not be applicable to the extraction of mouse melanocytes from adult mouse skin tissue because most of the mouse melanocytes are distributed in hair follicles that are located in the skin dermis. With the aim of efficient digestion of the dermis area, we used collagenase, which may increase the rate of dissociation of the melanocyte population from the mouse skin.

**Fig. 2:**
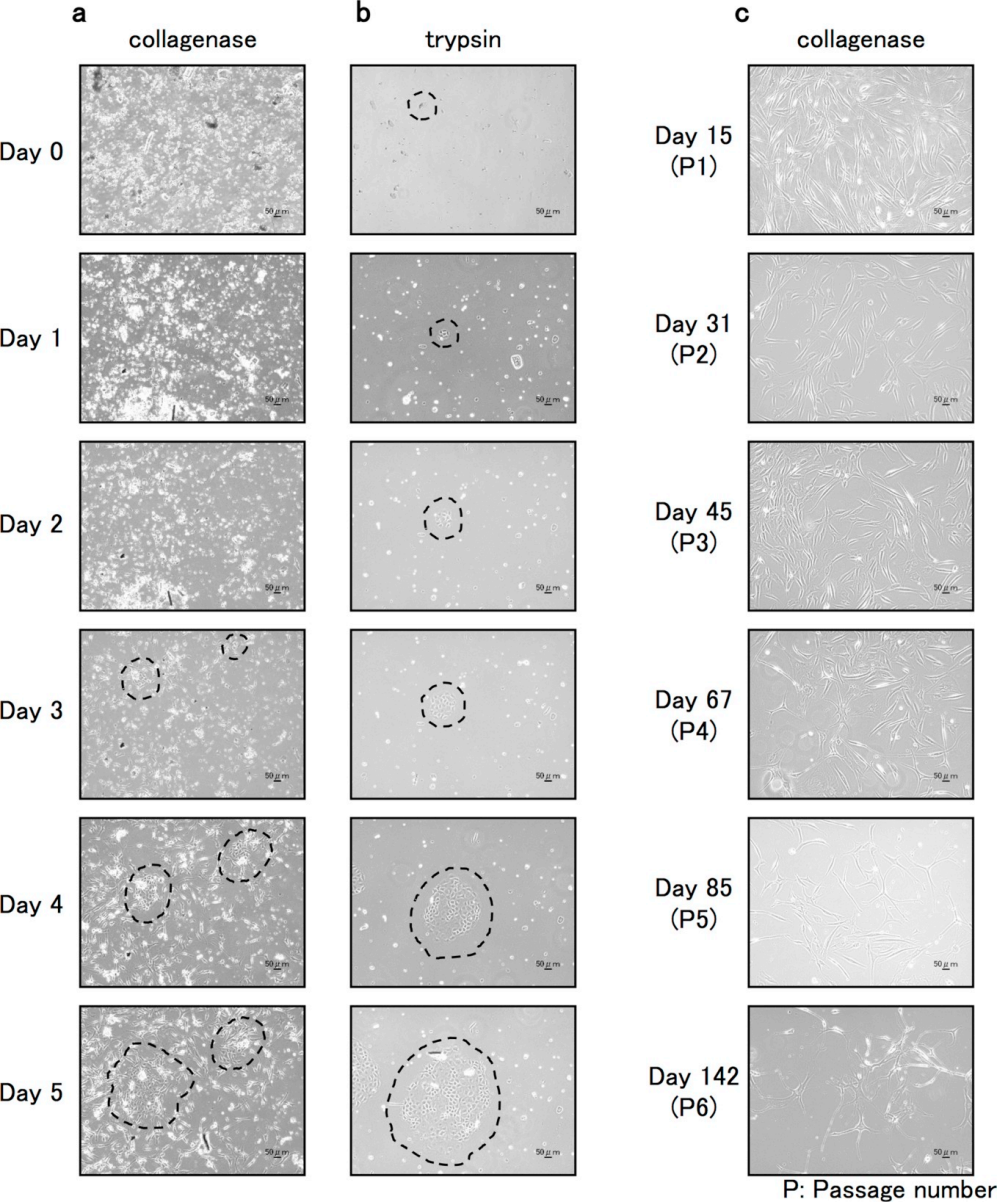
Observation of cell conditions (morphology, cell contamination, growth and cell density) after skin cell extraction by collagenase (a and c) and trypsin (b) at regular intervals. The extracted cells attached to the culture dish within 1 day. On Day 4 and Day 5 without subculturing, melanocyte-like cells with long protrusions were dominant with the collagenase method, while keratinocyte-like cells with polygonal morphology were mainly observed with the trypsin method. The encircled areas with a dotted line show the keratinocyte-like cell population. Bars represent 50 μm.

First, we prepared mouse skin tissue and cut the tissue with scissors into pieces of about 3 mm in diameter (Fig. 1). The chopped specimens were treated with either collagenase or trypsin. After removal of the digested skin debris from each treated sample, the dissociated cells were collected by centrifugation. At that time, we noticed that the number of extracted cells was much larger with collagenase treatment than with trypsin treatment, suggesting more efficient digestion of the skin specimen with collagenase than with trypsin. The cells were then cultivated with a medium specialized to normal human melanocytes. This specialized medium is good for cultivation of melanocytes. However, the medium is not adapted to selective propagation of a melanocyte population from a mixed cell condition that includes mainly keratinocytes and fibroblasts, which exhibit higher growing potential in culture. We hence supplemented the medium with TPA and cholera toxin. TPA and cholera toxin are effective for suppressing growth of contaminated keratinocytes and fibroblasts, respectively, without harmful effects on melanocytes [10,12]. By using the modified medium, we started the primary culture. Interestingly, in the collagenase-treated sample, there were many melanocyte-like cells with elongated protrusions like neuronal cells that were clearly different from the shape of fibroblasts and keratinocytes on culture Day 4 and Day 5 (Fig. 2a). The mixed cell culture also included keratinocyte-like cell populations but not fibroblast-like cell populations. On the other hand, in the trypsin-treated sample, only keratinocyte-like populations were appeared as clear colonies on Day 4 and Day 5 (Fig. 2b). A similar phenomenon was also observed when we used a third digestion medium that includes both collagenase and trypsin enzymes for the first procedure of mouse skin digestion (data not shown), probably due to cleavage of collagenase by trypsin, leading to inactivation of collagenase. The results indicate that single treatment with collagenase enables efficient extraction of melanocytes from adult mouse skin tissue.

### 3.2 Selective propagation of a melanocyte-like cell population in culture

**Fig. 3:**
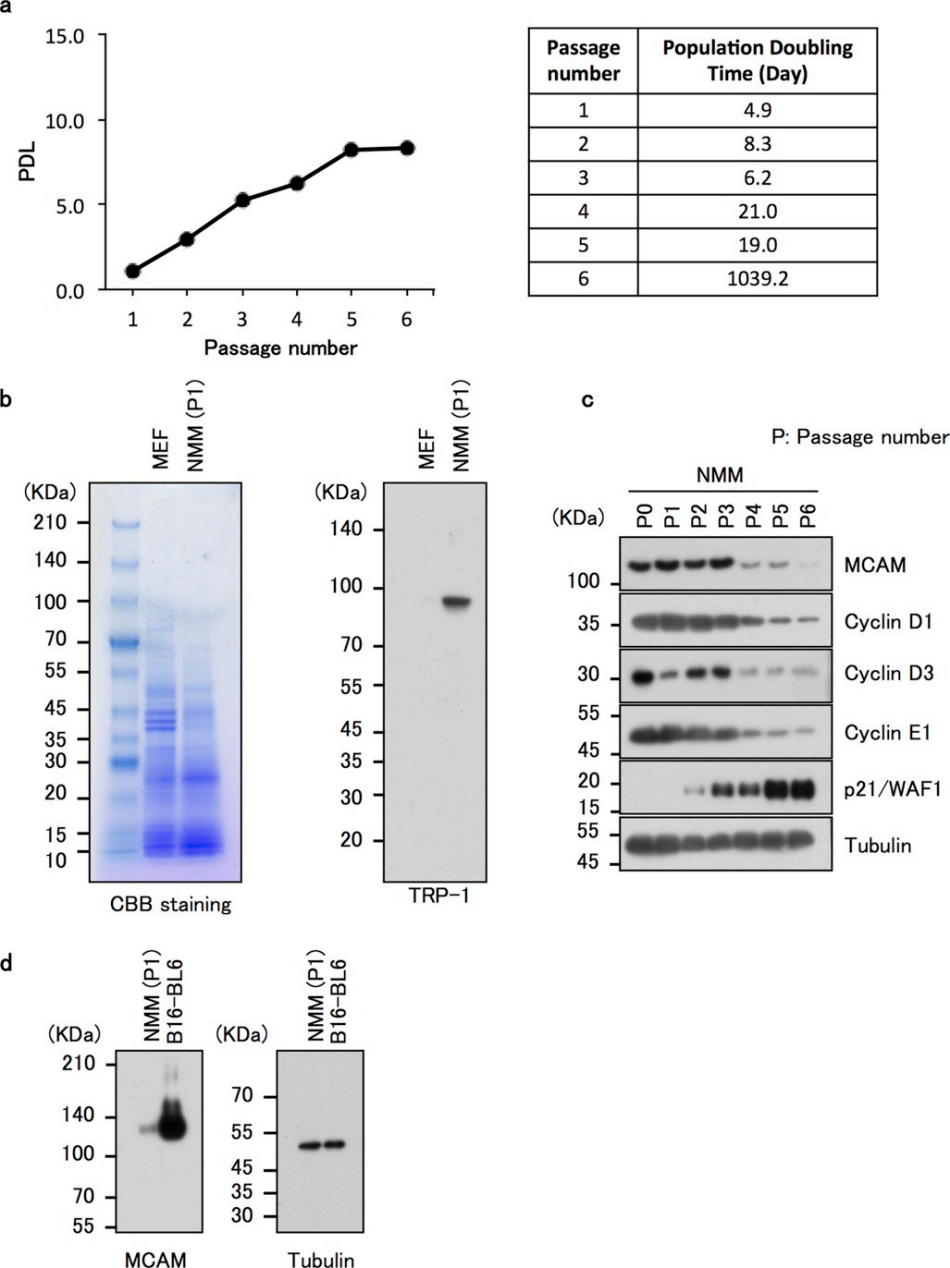
Analysis of the characteristics of the enriched mouse melanocyte-like cells. a, Level of cellular growth was monitored and plotted as population doublings against days (left panel). The population doubling times of cells that correspond to the indicated passage numbers (1–6) were displayed on the right panel. b, Mouse melanocyte-like cells at passage 1 (P1) were tested for their expression of TRP1, a representative melanocyte marker, by Western blot analysis using an anti-TRP1 antibody. Coomassie Brilliant Blue (CBB) staining was performed to check the proper loading of protein samples and for an internal control of the loaded proteins. MEF: mouse embryonic fibroblasts. NMM: normal mouse melanocytes. c, The enriched mouse melanocytes harvested at several passages were subjected to Western blotting for detection of MCAM and cell cycle-relevant proteins (cyclin D1, cyclin D3, Cyclin E1 and p21/WAF1). d, The enriched mouse melanocytes harvested at passage 1 (P1) were tested for their expression of MCAM in comparison to that in B16-BL6 mouse melanoma cell line by Western blot analysis.

To remove as many contaminated keratinocytes as possible from the collagenase-treated culture, we performed selective dissociation of melanocyte-like cells with trypsin on Day 5, when a time lag of detachment between melanocyte-like cells (weak attachment) and keratinocytes (tight attachment) occurred. In order to leave the keratinocyte population on the dish, we treated the cells for a short time under observation with a phase contrast microscope. By using the time-lag-based trypsinization method, we succeeded in obtaining an enriched melanocyte-like population with only one subculturing (Day 15) (Fig. 2c). The population-doubling level (PDL) of the cells was monitored and the resulting data was shown in Fig. 3a and it was possible to extend primary culture to 6 passages until cell longevity ceased.

### 3.3 Analysis of the characteristics of enriched mouse melanocyte-like cells

To determine whether the enriched melanocyte-like cells in culture were real melanocytes, cells at passage 1 (P1) were collected and subjected to Western blot analysis for detection of a representative melanocyte marker, tyrosinase-related protein-1 (TRP-1). We found that the propagated cells express TRP-1 at a pronounced level (Fig. 3b). Using the validated melanocyte population at the indicated passage numbers (Fig. 3a), we next examined the expression levels of cell cycle-related proteins. The cell cycle accelerators, cyclin D1, D3 and E1 were detected with significant levels at younger passages (P0-P3) and then they were all downregulated at the increased passages just starting from P1 through P6, while the expression of a representative cell cycle inhibitor, p21/WAF1 exhibited an inverse patterns to those of cyclins (Fig. 3c). We finally examined the expression level of MCAM in mouse B16-BL6 melanoma cells in comparison to that in normal mouse melanocytes at passage 1. As shown in Fig. 3d, we confirmed that the expression of MCAM is markedly higher in B16-BL6 melanoma cells than in normal cells. Interestingly, in normal cells, although MCAM was highly expressed in younger cells (P0-P3), it was markedly reduced in the older cells (P4-P6) like cyclines (Fig. 3c). These results suggest that MCAM plays a significant role in regulation of cellular growth or senescence in normal melanocytes. Thus, we succeeded in obtaining a convenient protocol for selective propagation of normal mouse melanocytes that is useful for several scientific aims.

In this protocol, we learned mainly three tricks, *i.e.*, use of collagenase for digestion of an adult mouse skin specimen, short trypsinization for subculturing, and use of TPA and cholera toxin to overcome the obstacle of contamination of keratinocytes and fibroblasts [13]. TPA is known to support the proliferation of normal human melanocytes in culture, but it causes growth suppression and rapid differentiation of keratinocytes [14]. In addition, TPA acts to prevent attachment of keratinocytes to the culture dish after trypsinization [15]. We hence considered that TPA and short trypsinization cooperatively cause the disappearance of contaminating keratinocytes from the primary culture. This may be the main reason for effective removal of keratinocytes in the primary culture. We also used cholera toxin, an adenylate cyclase activator, to prevent fibroblasts contamination. Although cholera toxin is useful for optimal proliferation of normal human melanocytes like TPA [10,11,16], it functions to prevent fibroblast proliferation since an intracellular increase in cyclic AMP produced by the activated adenylate cyclase enzyme efficiently blocks DNA synthesis of fibroblasts [16,17]. Considering the disappearance of fibroblasts in the primary mixed culture at a very early stage, the use of cholera toxin may greatly contribute to the removal of fibroblasts. When we searched for reports of similar methods, we found that Sviderskaya et al., top researchers in the field of melanocytes, had reported the beneficial role of TPA and cholera toxin for the primary culture of normal mouse melanocytes, which were from trypsinized embryonic mouse skin tissue [18]. We hence believe that our convenient protocol is firmly reliable as an experimental procedure for providing mouse melanocytes from adult mouse skin tissue. Lastly, for removal of the fibroblast population, other than cholera toxin, the antibiotic geneticin (G418 sulfate) may be effective since it was reported that treatment of a mixed primary culture from a human skin specimen with G418 at a concentration of 100 μg/ml for 2 days resulted in pure culture of normal human melanocytes [15].

## 4 Conclusion

In this study, our convenient method enabled the preparation of a pure population of normal mouse melanocytes in a culture system, which is very useful for comparison of cellular behaviors, alteration in the expression of genes and proteins, and metabolic alteration between mouse melanoma cells and their normal counterparts. The protocol may also be useful for young scientists who are doing research in fields related to melanocytes since, unlike human melanocytes, there is little information on normal mouse melanocytes due to the small number of reports on mouse melanocytes.

## Conflicts of interest

The authors declare that they have no conflicts of interest.

## Funding

This work was supported by grants from the JSPS KAKENHI Grant (No. 17H03577) (M. Sakaguchi) and Takeda Science Foundation (M. Sakaguchi).
